# Cargo diffusion shortens single-kinesin runs at low viscous drag

**DOI:** 10.1038/s41598-019-40550-5

**Published:** 2019-03-11

**Authors:** John O. Wilson, David A. Quint, Ajay Gopinathan, Jing Xu

**Affiliations:** 1 Department of Physics, University of California, Merced, California 95343 USA; 2 NSF CREST: Center for Cellular and Biomolecular Machines, University of California, Merced, California 95343 USA

## Abstract

Molecular motors such as kinesin-1 drive active, long-range transport of cargos along microtubules in cells. Thermal diffusion of the cargo can impose a randomly directed, fluctuating mechanical load on the motor carrying the cargo. Recent experiments highlighted a strong asymmetry in the sensitivity of single-kinesin run length to load direction, raising the intriguing possibility that cargo diffusion may non-trivially influence motor run length. To test this possibility, here we employed Monte Carlo-based simulations to evaluate the transport of cargo by a single kinesin. Our simulations included physiologically relevant viscous drag on the cargo and interrogated a large parameter space of cytoplasmic viscosities, cargo sizes, and motor velocities that captures their respective ranges in living cells. We found that cargo diffusion significantly shortens single-kinesin runs. This diffusion-based shortening is countered by viscous drag, leading to an unexpected, non-monotonic variation in run length as viscous drag increases. To our knowledge, this is the first identification of a significant effect of cargo diffusion on motor-based transport. Our study highlights the importance of cargo diffusion and load-detachment kinetics on single-motor functions under physiologically relevant conditions.

## Introduction

Molecular motors such as kinesin-1 are mechanoenzymes that drive long-range transport of cargos in living cells^1,2^. This transport process is challenging to accomplish, because motors must overcome substantial thermal diffusion to maintain directional transport. Thermal diffusion encompasses the set of random, non-directional motions that result from thermal agitation^3^. Thermal diffusion plays important roles in a variety of biological processes, including early embryonic patterning^4,5^, cell signaling^6^, and metabolism^7^. For motor-based transport, thermal diffusion can manifest as random motions of the motor or of the cargo. A recent investigation highlighted a significant effect of thermal diffusion of individual motor domains on single-kinesin function *in vitro*^8^. How thermal diffusion of the cargo influences motor-based transport, however, has remained unclear. While previous numerical modeling^9^ did not uncover a significant effect of cargo diffusion on single-motor function, recent modeling work^10^ indicated that changing the solution viscosity significantly affects cargo navigation across three-dimensional microtubule intersections, suggesting a likely effect of cargo diffusion on motor function.

The functions of molecular motors are affected by external force, or “load”^11–13^. Until recently, kinesin-1 was thought to be affected by load oriented in the direction opposite (“hindering”) of motor motion, but not by load oriented in the same (“assisting”) direction. This notion was reflected in previous numerical modeling studies, including work that predicted a null effect of cargo diffusion on single-kinesin transport^9^. However, recent single-molecule investigations^12,13^ revealed a significant impact of assisting load on the distance traveled by a single kinesin (“run length”), revising our understanding of the dependence of single-kinesin function to load. Importantly, these recent studies demonstrate a strong and perhaps counterintuitive asymmetry in the effect of load on single-kinesin run length: under the same amount of load, kinesin’s run length is significantly *shorter* when the load is in the direction assisting versus hindering motor motion^12,13^. In the current study, we carried out the first investigation of how this asymmetric sensitivity combines with cargo diffusion to impact kinesin’s motor function.

Thermal diffusion of the cargo can exert load on the motor. Importantly, because cargo diffusion is not correlated with motor motion^14,15^, the direction of the load from cargo diffusion can assist or hinder motor motion, depending on whether the cargo is leading in front of or lagging behind the motor. Given the recently identified asymmetric response of kinesin run length to load direction^12,13^, we hypothesized that cargo diffusion may non-trivially influence the run length of the kinesin carrying that cargo.

Here we employed Monte Carlo-based simulations to numerically examine the effects of cargo diffusion on transport by a single kinesin. Our study builds on previous numerical models^9,16^ and incorporates the recently uncovered effect of assisting load on single-kinesin run length^12,13^. We carried out our simulations over a large parameter space that captures crucial transport characteristics in living cells, including variations in cytoplasmic viscosity^17–22^, cargo size^22–28^, and transport velocity^29,30^. Our simulations included the physiologically relevant viscous drag that is associated with these parameter choices. Our simulations revealed that cargo diffusion significantly shortens single-kinesin run length at low viscous drag; this diffusion-based shortening effect arises from the specific asymmetry in the response of kinesin run length to load direction.

## Results

### Thermal diffusion of the cargo shortens the run length of single-kinesin cargos

We used a previously developed Monte Carlo simulation^9,16^ to examine the effect of cargo diffusion on kinesin run length in a viscous medium (Methods). In this simulation, the motor steps directionally along the microtubule track, while its cargo undergoes both random thermal diffusion and deterministic drift under load^3,14,15^. The direction and the value of the load on the cargo and the motor are determined by the displacement between them. The effect of load on run length is modeled by the motor’s load-detachment kinetics (Methods), which describes the probability of the motor detaching from the microtubule per unit time (“detachment rate”) for a given load value and direction. Previously, this and similar numerical simulation models included kinesin’s load-detachment kinetics under hindering load only and assumed that the motor’s detachment rate is unaffected by assisting load^9,16^. In the current study, we extended the load-detachment kinetics of the simulated motor (Methods) to reflect recent experimental measurements of the motor’s detachment rate under load oriented in both the assisting and the hindering directions^12,13^.

We first examined the run length of single-kinesin cargos over a physiologically relevant range of solution viscosities^17–22^, while holding cargo size and motor velocity constant at 0.5 µm in diameter and 0.8 µm/s when unloaded, respectively. These values are commonly captured in *in vitro* studies and are within the ranges measured for intracellular cargos^22–30^.

**Figure 1:**
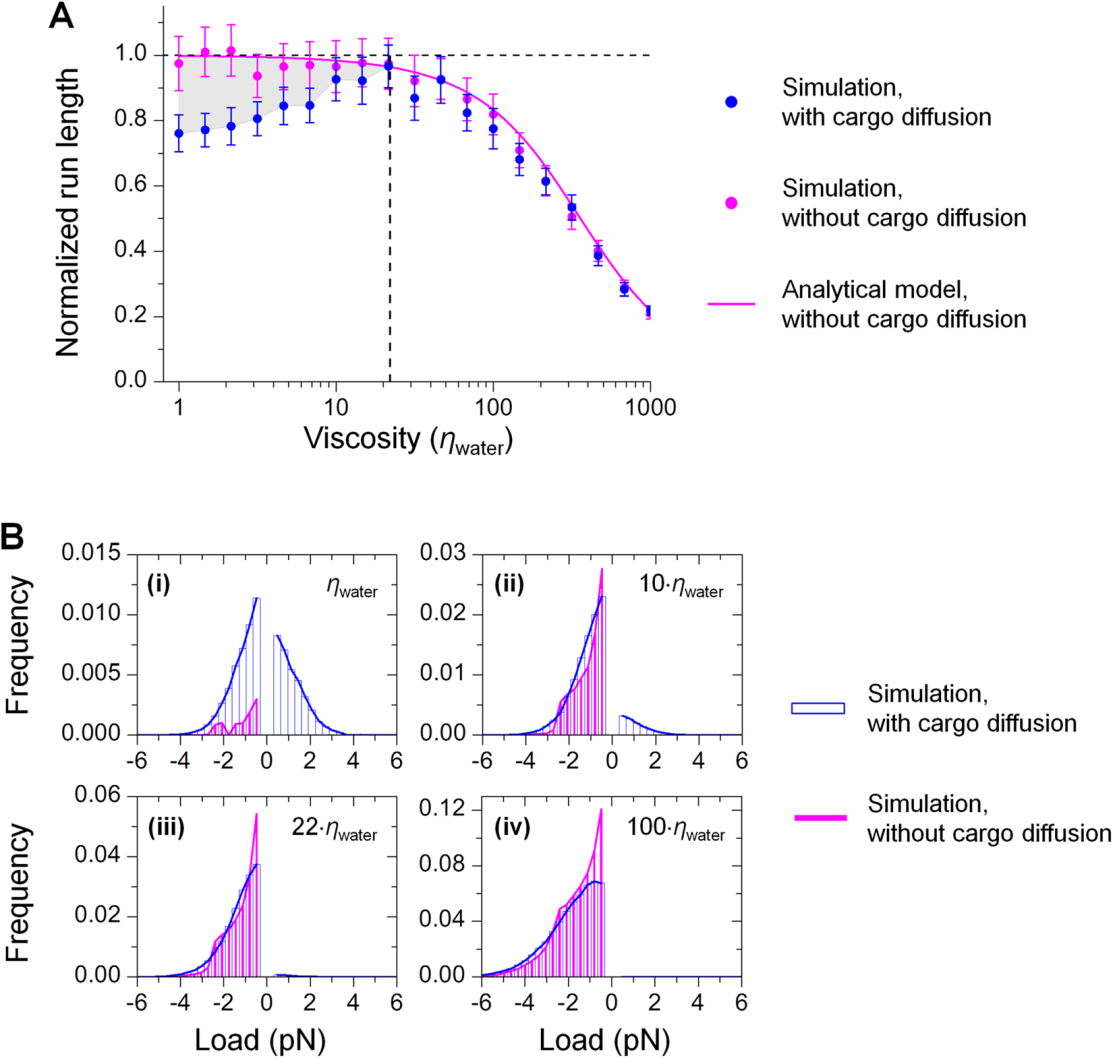
Cargo diffusion shortens single-kinesin run length at low viscosities (**A**) by imposing substantial assisting load on the motor (**B**). Simulations were carried out using a cargo 0.5 µm in diameter and a motor velocity of 0.8 µm/s unloaded. *η*_water_, the viscosity of water. (**A**) Run length (mean ± standard error of the mean) was normalized by the unloaded single-kinesin run length. *N* = 1000 for each simulation condition. Grey area, the difference in run length between simulations with and without cargo diffusion. Vertical dashed line, a viscosity 22-fold higher than that of water (22·*η*_water_). (**B**) Thermal diffusion of the cargo increases the load on the motor at low viscosities. Positive values indicate load in the direction that assists motor movement; negative values indicate load in the direction that hinders motor movement.

Perhaps surprisingly, our simulations revealed a non-monotonic dependence of run length on solution viscosity (blue scatters, Fig. 1A). Whereas the mean run length reached only 76 ± 6% of the unloaded single-kinesin value at the viscosity of water, it recovered to 97 ± 7% of the unloaded single-kinesin value at a viscosity ~22-fold higher than that of water, before declining with further increases in solution viscosity (blue scatters, Fig. 1A). In contrast, when we did not include thermal diffusion of the cargo in our simulations, we detected only a simple monotonic effect of viscosity on run length; importantly, run length remained approximately the same as the unloaded single-kinesin value at low viscosity (magenta scatters, Fig. 1A). Our simulations of the diffusion-free case were in excellent agreement with predictions of the analytical model that considers the motor’s response to viscous load but not cargo diffusion (Methods) (magenta line, Fig. 1A). The reduction in run length for simulations carried out in the presence of cargo diffusion versus the diffusion-free case was pronounced at low viscosity (grey area, Fig. 1A). This difference in run length vanished at higher viscosities, where viscous drag alone was sufficient to shorten cargo runs (magenta, Fig. 1A).

Together, our data demonstrate that thermal diffusion of the cargo results in kinesin run lengths that are shorter than those achieved without diffusion. This effect is localized to the low-viscosity range (grey area, Fig. 1A), yielding a non-monotonic dependence of run length on solution viscosity.

### Cargo diffusion imposes assisting load on the motor that is absent in the diffusion-free case

How does cargo diffusion shorten single-kinesin run length? Molecular motors such as kinesin are affected by mechanical load; a shorter run length suggests a larger load on the motor^11–13^. We thus hypothesized that cargo diffusion increases the load on the motor, particularly at the low viscosities at which we detected substantial diffusion-based shortening (grey area, Fig. 1A). To test this hypothesis, we compared the distribution of load on the motor between simulations with and without cargo diffusion.

We found that cargo diffusion introduced substantial assisting load on the motor at low viscosities (positive load, blue, Fig. 1B,i–iii). For example, at the viscosity of water, the motor had a similar probability of experiencing load in the assisting direction as in the hindering direction (positive vs. negative load, blue, Fig. 1Bi). In contrast, in the diffusion-free case, the motor experienced load only in the hindering direction (negative load, magenta, Fig. 1Bi), which is expected because viscous drag always opposes cargo motion. Note that cargo diffusion also increased the hindering load on the motor at low viscosity. For example, at the viscosity of water, the motor had a higher probability of experiencing a greater hindering load in the presence of cargo diffusion than in the diffusion-free case (negative load, blue vs. magenta, Fig. 1Bi). This observation is reasonable: thermal diffusion of the cargo is not correlated with the direction of motor motion^3^ and can thus contribute to load in both directions. As viscosity increased, the difference in load distributions diminished more quickly in the hindering direction than in the assisting direction (negative load vs. positive load, Fig. 1B,i–iii).

Taken together, our data demonstrate that cargo diffusion imposes substantial assisting load on the motor at low viscosities. Because assisting load *shortens* kinesin’s run length more severely than does hindering load^12,13^, diffusion-based assisting load supports the observed reduction in run length versus the diffusion-free case (grey area, Fig. 1A).

### The effect of cargo diffusion on run length depends non-monotonically on viscous drag

We next sought to understand how cargo size and/or motor velocity impact the run length of single kinesins carrying a cargo. While these parameters were held constant in the preceding simulations at 0.5 µm in diameter and 0.8 µm/s unloaded, respectively (Fig. 1), their values are known to vary in living cells^22–30^.

**Figure 2:**
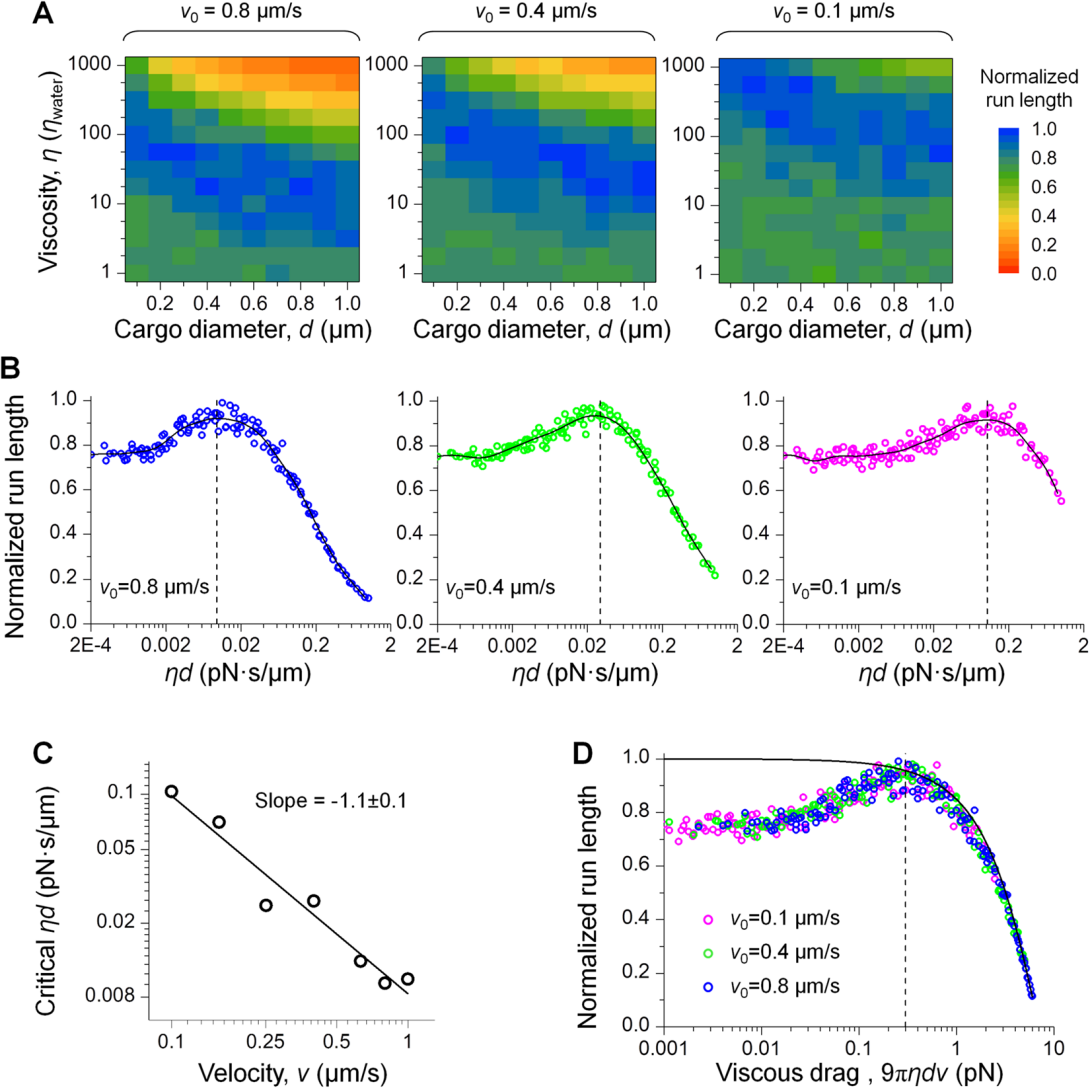
Non-monotonic variation in run length is general for physiologically relevant ranges of cargo size and motor velocity (**A**) and is summarized by the single parameter of viscous drag (**B**–**D**). *v*_0_ indicates the unloaded single-motor velocity. Run length was normalized by the unloaded single-motor value. *N* = 1000 for each simulation condition. (**A**) Dashed line, example inverse relationship between viscosity and cargo size as a guide to the eye. *η*_water_, the viscosity of water. (**B**) For each unloaded motor velocity, the impact of solution viscosity and cargo size on run length (panel A) is summarized as that of their product *ηd*. Solid line, smoothed moving average of simulated run lengths to guide the eye. Vertical dashed line, critical *ηd* value, where run length approaches that of the unloaded single kinesin. (**C**) The critical *ηd* value scales inversely with motor velocity. Solid line, best linear fit with the indicated slope. (**D**) The impact of *ηd* and motor velocity (*v*) on run length (panel B) is summarized as that of viscous drag (9π*ηdv*). Solid line, model prediction of run length for the diffusion-free case. Vertical dashed line, an approximate threshold (0.3 pN) where the shortening effect of viscous drag on kinesin run length exceeds 5% of the unloaded single-kinesin value.

We first examined the impact of cargo size, while holding motor velocity constant at 0.8 µm/s unloaded. The effect of solution viscosity on run length remained non-monotonic for cargos 0.1–1 µm in diameter (*v*_0_ = 0.8 µm/s, Fig. 2A). Interestingly, the viscosity at which run length most closely approached the unloaded single-motor value (“critical viscosity”) scaled inversely with cargo size (Fig. 2A, left). Because viscosity (*η*) and cargo size (*d*) enter the problem via viscous drag on the cargo, which scales as the product *ηd*, a reasonable ansatz would be for run length to depend on this product. Consistent with this hypothesis, the simulated run lengths for each combination of solution viscosity and cargo size collapsed onto a single curve with *ηd* as the control parameter (Fig. 2B, left).

We next examined the impact of motor velocity on our simulation results. For each unloaded motor velocity examined, the run length of single-kinesin cargos again varied non-monotonically with the combined parameter *ηd* (Fig. 2A,B, middle and right). Interestingly, the value of *ηd* at which run length approached the unloaded single-motor value correlated inversely with motor velocity (Fig. 2C). This inverse scaling suggests that the effects of *ηd* and motor velocity (*v*) on run length may be again combined as that of their product *ηdv*^31^, or equivalently the viscous drag experienced by the cargo (modeled as 9π*ηdv*, see Discussion). Consistent with this hypothesis, the run length for the three unloaded motor velocities (Fig. 2B) collapsed onto a single curve with viscous drag as the single control parameter (Fig. 2D).

Thus, our simulations demonstrate that the run length of single-kinesin cargos is influenced by three independent parameters: solution viscosity, cargo size, and motor velocity. The effect of these three parameters on run length is summarized as that of a single control parameter: the product of the three parameters, or viscous drag that arises from the active motion of the motor. This collapsed single-parameter curve differs substantially from model predictions for the diffusion-free case at low viscous drag (≤0.2 pN, Fig. 2D). This difference diminishes when the effect of viscous drag on kinesin’s run length becomes pronounced (scatters vs. solid line, Fig. 2D).

### Viscous drag biases thermal diffusion of cargo toward the hindering direction

We next sought to understand the impact of viscous drag on the displacement of the diffusing cargo from the motor; this displacement information is important because it determines the load on the motor.

**Figure 3:**
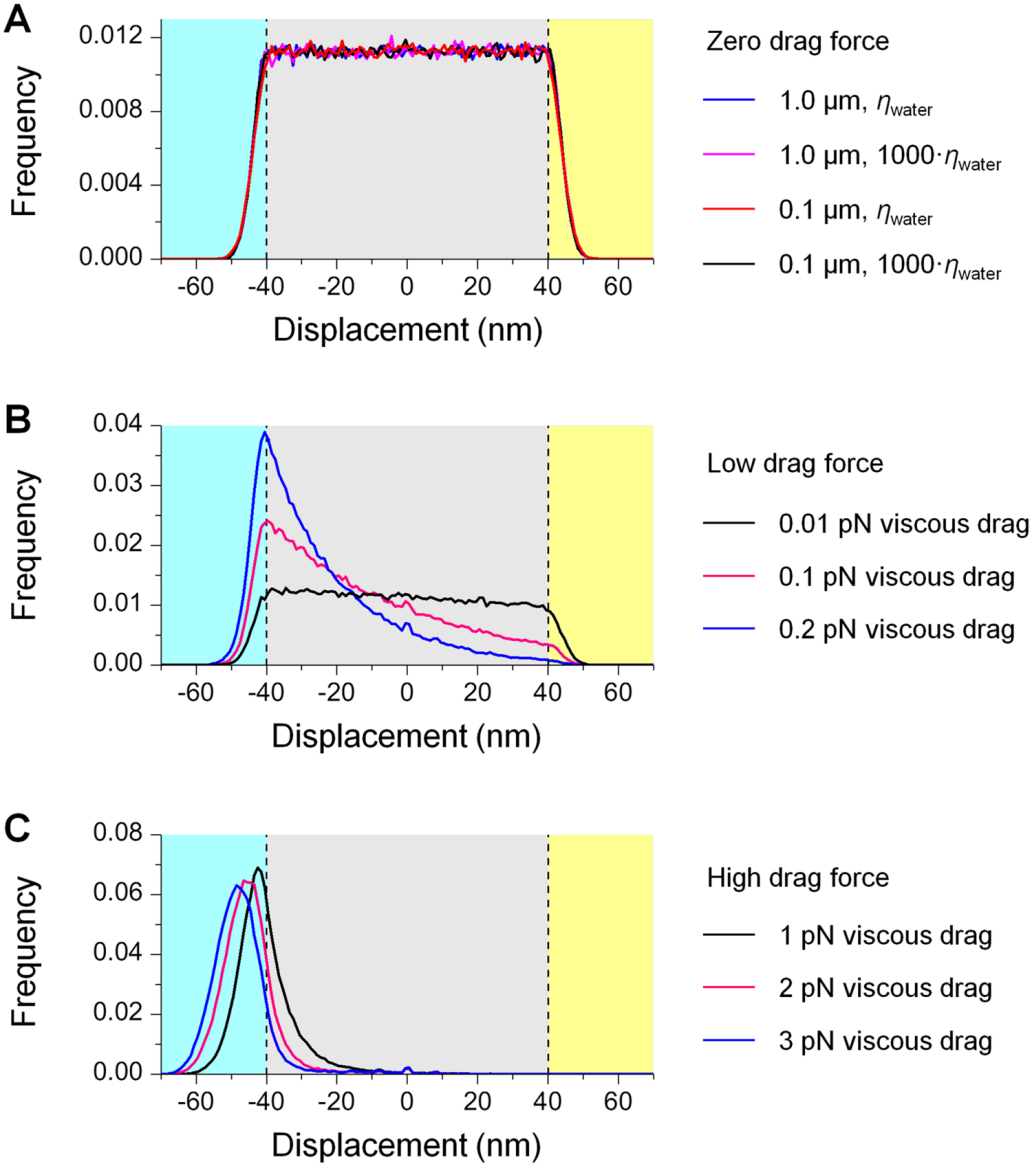
Distributions of the displacement of thermally diffusing cargo from the motor, simulated at zero viscous drag (**A**), low viscous drag (**B**), and relatively high viscous drag (**C**). Positive displacement reflects the cargo leading in front of the motor; negative displacement indicates that the cargo lags behind the motor. Grey area, free-diffusion range where the cargo does not impose load on the motor. Cyan (and yellow) area, tethered-diffusion range where the cargo imposes hindering (and assisting) load on the motor. (**A**) At zero viscous drag, the displacement of the diffusing cargo is symmetric about the motor position (0 nm) and is not sensitive to cargo size or solution viscosity. *N* = 10 for each simulation condition, with each simulation including 20,000 times steps. (**B**,**C**) At both low (**B**) and relatively high (**C**) viscous drag, the displacement of a diffusing cargo is biased toward the hindering direction (negative displacement). The extent of this bias increases as the viscous drag increases. *N* = 1000 for each simulation condition.

**Figure 4:**
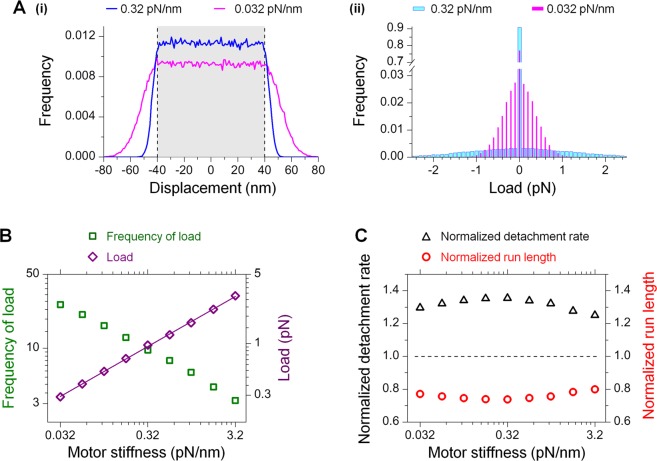
The effect of cargo diffusion on single-kinesin run length is not strongly influenced by motor stiffness. Simulations were carried out at zero viscous drag. *N* = 10 for each simulation condition, with each simulation including 20,000 times steps. (**A**) Motor stiffness impacts (i) the displacement of the diffusing cargo from the motor and (ii) the resulting load on the motor. Grey area, free-diffusion range where the cargo does not impose load on the motor. (**B**) Increasing motor stiffness decreases the frequency with which the diffusing cargo imposes load on the motor (green squares), while increasing the mean magnitude of the load on the motor (purple diamonds). Solid line, best linear fit with a slope of 0.505 ± 0.004. (**C**) The motor’s detachment rate (black triangles) and run length (red circles) were largely unchanged over the range of motor stiffness tested. Detachment rate and run length were normalized by their unloaded single-motor values.

We first carried out simulations for the case of zero viscous drag (Fig. 3A). Here, the motor velocity was set at 0 µm/s to realize a zero drag force, and solution viscosity and cargo size were varied over the physiologically relevant ranges used in preceding simulations (1000-fold and 10-fold ranges, respectively). The resulting displacement distributions were symmetric about the motor position and exhibited two diffusion regimes: a uniformly distributed “free diffusion” range (grey area, Fig. 3) where thermal motion of the cargo does not stretch the motor beyond its rest length (Methods) and is thus effectively decoupled from the motor; and a normally distributed “tethered diffusion” range (cyan and yellow areas, Fig. 4) where thermal excursion of the cargo is restricted by the motor that tethers the cargo to the microtubule^15^. Displacement distributions were not sensitive to cargo size or solution viscosity, with each distribution demonstrating a similar probability and a similar mean excursion of the cargo in the tethered diffusion range (~5% and ~3 nm, respectively and in both load directions, cyan and yellow areas, Fig. 3A). These displacement distributions correspond to a 30% increase in the motor’s detachment rate and a 26% reduction in motor run length from their unloaded values (Methods). These values are in excellent agreement with the ~24% reduction in run length in our simulations at negligible viscous drag (1 × 10^−3^ pN, Fig. 2D).

We next examined the case of low viscous drag (Fig. 3B). Here, the motor velocity was kept constant at 0.8 µm/s, and solution viscosity and cargo size were chosen to capture the low viscous drag range that alleviates the shortening effect of cargo diffusion on kinesin run length (0.01–0.2 pN, Fig. 2D). Within this force range, the effect of viscous drag on kinesin run length was ≤3% of the unloaded single-kinesin value (solid line, Fig. 2D). The resulting displacement distributions were asymmetric about the motor position in both the free-diffusion range (grey area, Fig. 3B) and the tethered-diffusion range (cyan and yellow areas, Fig. 3B). As the viscous drag increased, the position of the diffusing cargo increasingly lagged behind the motor. At a drag force of 0.2 pN, the probability that the cargo will exert load in the assisting direction diminished to <0.4% (blue line, Fig. 3B). Of note, despite the asymmetry in the displacement distribution, mean excursion of the cargo in the tethered-diffusion range remained similar between load directions (~2.9 nm in the hindering direction and ~3.2 nm in the assisting direction, cyan and yellow areas, Fig. 3B) and similar to that for zero viscous drag (~3 nm in both load directions, cyan and yellow areas, Fig. 3A). For comparison, at higher viscous drag (1–3 pN, Fig. 3C), the displacement of the diffusing cargo was further biased toward the hindering direction (cyan area, Fig. 3C); the mean excursion of the cargo in the hindering direction increased as the viscous drag increased (cyan area, Fig. 3C).

Thus, our simulations indicate that viscous drag biases the diffusing cargo to lag behind the moving motor, which reduces the probability of the motor experiencing assisting load. At low viscous drag, this reduction in assisting load is accompanied by an increased probability, but not the magnitude, of hindering load on the motor.

### The effect of cargo diffusion on run length is not strongly influenced by motor stiffness

Because the stiffness of the motor is a key determining factor for tethered diffusion^9,14,15^, we hypothesized that the effect of cargo diffusion on run length may be influenced by motor stiffness. We carried out simulations at zero viscous drag to test this possibility. As experimental measurements of the stiffness of molecular motors (or other proteins) are still limited, here we examined a large, 100-fold range of values of motor stiffness, including available *in vitro* experimental measurements for single-kinesin transport^32^ and multiple-motor transport^33–35^.

Our simulations demonstrate that although motor stiffness impacts both the probability and the extent of cargo displacement in the load-imposing, tethered-diffusion range (Fig. 4A,B), these two factors do not combine to substantially alter the effect of cargo diffusion on single-kinesin run length (Fig. 4C). As the motor linkage increased in stiffness, there was a higher probability of the cargo remaining in the free-diffusion range (grey area, Fig. 4Ai; 0 pN, Fig. 4Aii), and a lower probability of the cargo diffusing in the tethered range to exert load on the cargo (green, Fig. 4B). These observations are expected for tethered diffusion^9,14,15^. On the other hand, the magnitude of the load from the cargo increased as motor stiffness increased (blue vs. magenta, Fig. 4Aii, and purple diamonds, Fig. 4B), varying as the square root of motor stiffness as expected from equipartition theorem in statistical physics^14,31^ (solid line, Fig. 4B). Thus, the stiffness of the motor has opposite effects on the probability of the cargo imposing load on the motor (green squares, Fig. 4B) and the magnitude of the load that the cargo can impose (purple diamonds, Fig. 4B). Over the 100-fold range of motor stiffnesses tested, these two opposing effects resulted in a modest, 3.5% change in the motor’s detachment rate (black triangles, Fig. 4C), corresponding to a similarly modest, 3.8% change in run length over the same stiffness range (red circles, Fig. 4C).

Taken together—and contrary to our initial expectation—our data indicate that the effect of cargo diffusion on single-kinesin run length is not strongly influenced by motor stiffness.

### Non-monotonic variation in run length requires specific asymmetry in the motor’s load-detachment kinetics

**Figure 5:**
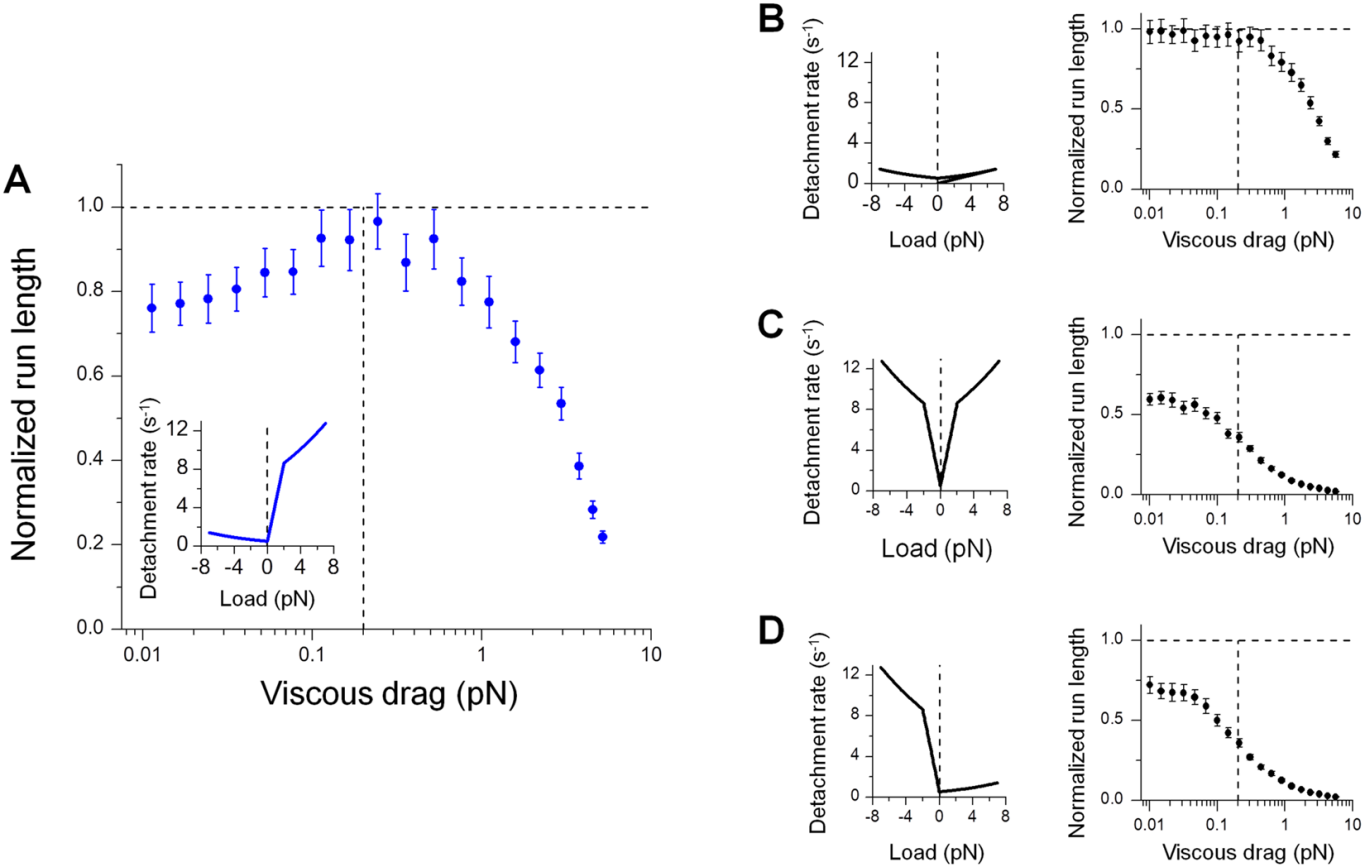
Non-monotonic dependence of run length on viscous drag reflects a specific asymmetry in the motor’s load-detachment kinetics. Vertical dashed lines in run-length panels, the critical viscous drag force (0.2 pN, Fig. 2D) that separates the region where the effect of thermal diffusion dominates (left) from the region where the effect of viscous drag dominates (right). (**A**) Experimentally measured load-detachment kinetics^12,13^ (inset) give rise to a non-monotonic dependence of run length on viscous drag. Data are duplicated from Fig. 1A (blue scatter). (**B**,**C**) Symmetric load-detachment kinetics (left) cannot support a non-monotonic dependence of run length on viscous drag (right). (**D**) Asymmetric load-detachment kinetics with reduced sensitivity for assisting versus hindering load (left) also cannot support a non-monotonic dependence of run length on viscous drag (right). For all panels, run length (mean ± standard error of the mean; *N* = 1000 for each simulation condition) was normalized by the unloaded single-kinesin value.

We next sought to understand how specific asymmetry in kinesin’s load-detachment kinetics influences run length behavior. To address this, we varied the symmetry properties of the motor’s load-detachment kinetics under otherwise identical simulation conditions. We duplicated our preceding simulations and the associated experimentally measured load-detachment kinetics for single kinesins^12,13^ for ease of comparison (Figs 1A and 5A).

We found that asymmetry in kinesin’s load-detachment kinetics is necessary but not sufficient for the observed non-monotonic dependence of run length on viscous drag (Fig. 5B–D). We first examined the effect of symmetric load-detachment profiles on run length (Fig. 5B,C). Here, we duplicated the experimentally measured load dependence^12,13^ in the hindering direction (left, Fig. 5B) or the assisting direction (left, Fig. 5C). In both cases, the effect of viscous drag on run length increased monotonically, with run length maintaining its maximum value at the lowest viscous drag tested (right, Fig. 5B,C). As expected, the maximum run length of the single-motor cargo was substantially shorter when we assumed a higher sensitivity of the motor’s detachment rate to load (right, Fig. 5C,B). We next implemented an asymmetric load-detachment profile that reversed the directional bias of kinesin’s load dependence (left, Fig. 5D); we again observed a monotonic dependence of run length on viscous drag (right, Fig. 5D).

Hence, our simulations reveal that the specific asymmetry in the load-detachment kinetics of kinesin—steeper sensitivity for assisting versus hindering load^12,13^ (inset, Fig. 5A)—underlies the non-monotonic dependence of run length on viscous drag uncovered in the current study.

## Discussion

Here we used Monte Carlo-based simulations to examine the effect of thermal diffusion of the cargo on the run length of a single kinesin carrying the cargo. To our knowledge, this is the first identification of a significant effect of cargo diffusion on motor-based transport. We found that cargo diffusion shortens single-kinesin runs by imposing substantial load in the direction of transport; this load is absent in the diffusion-free case. This diffusion-based shortening is countered by viscous drag, which biases the effect of the diffusing cargo toward the hindering load. Combined, our simulations revealed an unexpected, non-monotonic variation in run length, which is impaired at low and high viscous drag, but recovers to the unloaded single-motor value at intermediate viscous drag. We determined that the shortening effect of cargo diffusion on run length is not strongly sensitive to motor stiffness, and that the specific asymmetry in kinesin’s load-detachment kinetics underlies the non-monotonic variation of run length uncovered in the current study.

Our simulations reveal a novel, dual effect of viscous drag on molecular motor-based transport. Because viscous drag opposes cargo motion, it is generally examined in the context of impairing motor-based transport^36–38^. Consistent with this notion, we observed substantial impairment at high viscous drag (Fig. 2D). However, at lower viscous drag that does not significantly influence motor functions, our simulations indicate a novel, “recovery” effect of viscous drag on run length (Fig. 2D). The resulting non-monotonic variation in run length may be important for understanding the diverse characteristics of transport in living cells, where highly variable conditions can combine to alter viscous drag—and hence run length—non-trivially. Such predictions may be tested experimentally by combining fluorescence-based run length measurements^39,40^ with ~10-fold variations in solution viscosity^37,38^, cargo size^41^, and motor velocity^42^. This would allow one to achieve a 1000-fold variation in viscous drag needed to explore the full range of the non-monotonic variation of run length (Fig. 2D). Note that we modeled the viscous drag on the cargo as the Stoke’s drag near a hard wall (9π*ηdv*), which matches the experimental conditions for many *in vitro* studies but may not be appropriate for *in vivo* scenarios. Nonetheless, because our study identifies the *magnitude of viscous drag* as the single parameter controlling the impact of cargo diffusion on single-kinesin run length (Fig. 2D), we anticipate that the results of our study will hold for *in vivo* scenarios, even if the precise expression evaluating the drag force may be different.

An important implication of our study is that the specifics of load-detachment kinetics are likely critical for differentiating and fine-tuning the single-motor functions of distinct classes of motors under physiologically relevant conditions. The diffusion-based shortening of run length at low viscous drag arises from the motor’s sensitivity to assisting load (Fig. 1); the non-monotonic variation in run length with viscous drag reflects the specific asymmetry in the motor’s load-detachment kinetics (Fig. 5). The more likely the motor is to detach under load in the assisting versus the hindering direction, the greater the effect of cargo diffusion on shortening the motor’s run length, and the greater the non-monotonic variation in cargo run length with viscous drag. We thus predict similar non-monotonic variations in run length for other classes of motors whose detachment rates are more sensitive to assisting load than to hindering load, such as kinesin-2^43^ and cytoplasmic dynein^44^. The specifics of non-monotonicity in run length likely depend on the specific functional forms of their respective load-detachment kinetics. A potential sensitivity of the load-detachment kinetics to nucleotide concentrations, such as that experimentally identified^45,46^ and theoretically examined^47^ for the load-velocity dependence of kinesin-1, may drive further fine-tuning of single-motor functions *in vivo*.

Our findings at the single-molecule level are likely directly relevant for transport by small teams of kinesin-1, which is on average accomplished via the action of a single kinesin^48–50^. Thermal diffusion of multiple-motor cargos depends stochastically on the number of motors linking the cargo to the microtubule^33–35^. Because we did not detect a strong impact of motor stiffness on the shortening effect of cargo diffusion on run length (Fig. 4C), we speculate that the effects uncovered here may not be substantially altered by changes in effective stiffness in multiple-motor transport versus single-motor transport.

The effects uncovered here also highlight diffusion-based load as a new consideration for understanding multiple-motor transport, particularly for mixed classes of motors that differ in their load-detachment kinetics. Recent investigations have focused on the importance of inter-motor strain^35,51,52^ and local confinement^34,48^ on team-motor functions. The current study suggests that, depending on the specifics of the load-detachment kinetics of the motor(s) present, thermal diffusion of the cargo may preferentially shorten the run length of a particular class of motor engaged in team transport, a bias that may be further tuned by viscous drag. We are developing simulations to explore this intriguing possibility.

In summary, our simulations revealed a previously unexplored, non-monotonic variation of run length that arises from the interplay between cargo diffusion and solution viscosity. As an additional consideration, the elastic nature of the cytoplasm, which is strongly influenced by spatial heterogeneity of the cytoskeleton^53^, has been predicted to impact the velocity of a single, cargo-free kinesin^54^. Future investigations combining solution viscoelasticity with cargo diffusion may reveal additional diversity or tunability in cargo transport, for single motors and for multiple motors functioning in teams.

## Methods

### Monte Carlo-based simulation

A previously developed Monte Carlo-based simulation model^16^ was adapted to evaluate the transport of single-kinesin cargos in a viscous medium. The current study used the numerical algorithm developed previously^16^, but updated the motor’s load-detachment kinetics to reflect recent experimental data^12,13^ on the motor’s response to assisting load as well as the revisions to the previously considered response to hindering load.

Briefly, each cargo is carried by one motor that moves along a one-dimensional microtubule lattice. The motor is assumed to be an idealized spring with an unstretched, rest length and a linkage stiffness. The motor is assumed to experience a load only when the displacement between the motor and its cargo is larger than the motor’s rest length. A simulated cargo run is initiated when the motor becomes stochastically bound to the microtubule (characterized by the motor’s binding rate).

At each simulation time step, the displacement between the cargo and the motor is used to determine the load on the motor (and on the cargo). The load on the motor is used to determine the probability that the motor will detach from the microtubule (characterized by the motor’s load-detachment kinetics). If the motor remains engaged in transport, then the load on the motor is used to calculate the probability that the motor will advance one step along the microtubule lattice (characterized by the motor’s load-stepping kinetics), and the position of the motor is updated accordingly. The load on the cargo is used to determine the drift motion of the cargo that is tethered to the motor. When the cargo is subjected to a net force *F*, it moves through a viscous medium with a drift velocity *v*_drift_ = *F*/*ξ*, where *ξ* is the friction constant determined by the solution viscosity *η* and the diameter of the bead *d*: *ξ* = 3*πηd*. The net motion of the bead over the simulation time step (Δ*t*) is determined as the sum of this drift motion (*v*_drift_·Δ*t*) and the random thermal diffusion of the cargo. The simulation time step is incremented and the above evaluations are repeated until the motor stochastically detaches from the microtubule.

A simulation time step of 10 µs was used for all simulations for motor stiffness ≤0.32 pN/nm; this time step is faster than the typical time scale for the fastest process in the motor’s mechanochemical cycle^9^. For the cargo sizes examined in the current study (≥100 nm in diameter), no significant differences in simulation results were detected when we reduced our simulation time step to 1 µs (data not shown). Notably, for smaller cargos, a significantly faster simulation time step (for example, ≤0.6 µs for cargos 1 nm in diameter) is necessary to avoid overestimation of the diffusion distance of the cargo and hence the load on the motor within each simulation time step (data not shown).

A faster simulation time step of 10^−6^ s was used for simulations of stiffer motors (>0.32 pN/nm, Fig. 4), which better resolved the position of the cargo under higher tension from the stiffer motor linkage (data not shown).

A rest length of 40 nm was used for all simulations, reflecting the compact form of kinesin during transport^55,56^ and mitigating the difference between the idealized spring used in the simulation model^9,16^ and the previously reported strain-gated response of kinesin to small displacements between the motor and its cargo^57^.

Unless otherwise indicated, the same motor stiffness (0.32 pN/nm, refs ^32,58,59^), binding rate (5/s, ref.^60^), step size (8 nm, refs^42,61^), single-kinesin stall force (7 pN, ref.^12^), and unloaded run length (1.5 µm, ref.^48^) were used for all simulations. The values of solution viscosity, cargo size, and unloaded motor velocity are as indicated.

The viscous drag on the cargo was determined as 9π*dηv*, where *d* is the cargo diameter, *η* is the solution viscosity, and *v* is the velocity of the motor under viscous load. This expression describes the Stoke’s drag on a sphere near a hard wall, reflecting the experimental conditions for many *in vitro* studies.

The motor’s load-stepping kinetics was as determined in previous experimental^11,13,62^ and modeling^16^ studies:

$${k}_{step}(F)=\{\begin{array}{ll}0, & F\le -{F}_{s}\\ ({v}_{0}/{\rm{\Delta }}x)\cdot (1-{(F/{F}_{s})}^{2}), & -F{}_{s}\, < \,F\,\le 0\\ {v}_{0}/{\rm{\Delta }}x, & F > 0\end{array}$$

where *v*_0_ is kinesin’s unloaded velocity, Δ*x* is the motor’s step size, |*F|* is the magnitude of the load on the motor, and *F*_*s*_ is the single-kinesin stall force. Positive force indicates load in the direction assisting motor motion, and negative force indicates load in the direction hindering motor motion.

The motor’s load-detachment kinetics was as determined in recent experimental studies by Milic *et al*.^12^ and Andreason *et al*.^13^ for hindering forces between −25 pN and 0 pN, and for assisting forces between +2 pN and +20 pN. Extrapolation of measurements of these two ranges yields an apparent discontinuity^13^ in kinesin’s detachment rate at 0 pN. This apparent discontinuity has not yet been resolved experimentally: direct measurements of the motor’s load-detachment kinetics are not yet available for the 0–2 pN assisting force range. To mitigate this apparent discontinuity, here we modeled the detachment rate of kinesin as a linear continuation between available experimental measurements at 0 pN and at 2 pN^12,13^. Note that this linear-interpolation approach underestimates the effect of cargo diffusion uncovered in the current study. We summarize the motor’s detachment rate under load used in the current study as the piecewise function

$$\varepsilon (F)=\{\begin{array}{ll}{\varepsilon }_{0}\cdot \exp (|F|/{F}_{d-}), & F\le 0\\ {\varepsilon }_{0}\cdot (1+3.8247\cdot F),\, & 0 < F\le 2\\ {\varepsilon }_{0}\cdot 7.4\cdot \exp (F/{F}_{d+}), & F > 2\end{array}$$

where *ɛ*_0_ is the unloaded single-kinesin detachment rate, *F* is the load on the motor, *F*_*d−*_ is the detachment force of kinesin in the hindering direction, and *F*_*d*+_ is the detachment force of kinesin in the assisting direction. The unit of detachment rates is s^−1^, and the unit of forces is pN. All other numerical values are dimensionless. Positive force indicates load in the direction assisting motor motion, and negative force indicates load in the direction hindering motor motion. The unloaded detachment rate is determined as *ɛ*_0_ = *v*_0_/*l*_0_, where *v*_0_ is the unloaded single-kinesin velocity, and *l*_0_ is the unloaded single-kinesin run length. The value of the detachment force in the hindering direction was defined by Schnitzer *et al*.^11^ as *F*_*d−*_ = *k*_B_*T*/*δ*_*l−*_, where *k*_B_*T* is the thermal energy (4.11 pN·nm) and *δ*_*l−*_ is the characteristic distance between the attached and the detached states. The value of *δ*_*l−*_ was recently determined as 0.60 nm in Andreason *et al*.^13^, approximately half of the value previously reported by Schnitzer *et al*.^11^, likely reflecting the major technological advances in the force-clamping experiments used for these measurements^12,63,64^. The value of the detachment force in the assisting direction is similarly defined by Andreason *et al*.^13^ as *F*_*d*+_ = *k*_B_*T*/*δ*_*l*+_, where *δ*_*l*+_ = 0.32 nm.

### Data analysis

The run length of a simulated trajectory was defined as the overall distance traveled by the simulated motor before detaching from the microtubule. For each simulation condition, the cumulative probability distribution of the run lengths was fitted to the cumulative probability function of a single exponential distribution $1-A\cdot \exp (\,-\,x/l)$. Mean run length was determined as the best-fit decay constant *l*. The associated standard error of the mean was determined via a bootstrap method^65^.

The velocity of a simulated trajectory was determined as the best-fit slope of the trajectory. Only trajectories ≥0.2 s in duration were considered for analysis; of these trajectories, only those that moved ≥100 nm were analyzed. For each simulation condition, mean velocity was calculated as the mean of the normally distributed velocity values. The associated standard error of the mean was determined by a bootstrap method^65^.

The load on the motor for a given displacement of the cargo from the motor was determined as the length of the motor stretched beyond its rest value, multiplied by motor stiffness. The direction of the load was determined by the relative position of the cargo to the motor: “assisting” when the cargo position leads the motor, “hindering” when the cargo position lags behind the motor.

The effective detachment rate of the motor for a given distribution of displacements of the diffusing cargo from the motor (Figs 3 and 4) was determined as the weighted sum of kinesin’s detachment rate at a particular displacement value, multiplied by the frequency of occurrence of the particular displacement value. Kinesin’s detachment rate at a particular displacement value was calculated by first determining the load associated with the displacement value, then applying the motor’s load-detachment kinetics as described above. The run length was calculated as the ratio of cargo velocity to its detachment rate.

### Data representation

MATLAB functions colorcet.m^66^ and cmap2pal.m^67^ were used to generate the perceptually uniform colormap used in Fig. 2A.

### Analytical model of the run length of single-kinesin cargos in the diffusion-free case

In the absence of cargo diffusion, the only load on the motor is imposed by viscous drag in the direction that hinders the motor’s motion: |*F*| = 9π*dηv*, as described above for the Monte Carlo-based simulations.

The run length of single-kinesin cargos was determined as *l* = *v*/*ɛ*, where *v* is the velocity and *ɛ* is the detachment rate of the motor carrying the cargo. Based on the experimentally measured load-detachment kinetics of kinesin for hindering loads^11–13^ (described in the simulation model for *F* < 0), the run length of single-kinesin cargos is

$$l=\frac{v}{{\varepsilon }_{0}}\cdot \exp (\frac{-9\pi \eta dv}{{F}_{d-}}),$$

where *ɛ*_0_ is the unloaded single-kinesin detachment rate and *F*_*d−*_ is the single-kinesin detachment force under hindering load, as described above for the Monte Carlo-based simulations.

The velocity of the motor under viscous load in the preceding equation was calculated as follows. The experimentally measured load-velocity kinetics of kinesin for hindering loads^62^ is well approximated as^16^

$$v={v}_{0}\cdot (1-{(\frac{F}{{F}_{s}})}^{2}),$$

where *v*_0_ is the unloaded single-kinesin velocity, and *F* is the hindering load on the motor. The velocity of the motor under viscous load (|*F*| = 9π*dηv*) is then described as

$$v={v}_{0}\cdot (1-{(\frac{9\pi \eta dv}{{F}_{s}})}^{2}).$$

The solution to this above quadratic equation gives rise to the analytic description of the velocity of single-kinesin cargos in a viscous medium

$$v=\frac{{v}_{0}}{2}\cdot {(\frac{{F}_{s}}{9\pi d\eta {v}_{0}})}^{2}\cdot (-1+\sqrt{1+4{(\frac{9\pi \eta d{v}_{0}}{{F}_{s}})}^{2}}).$$

## Data Availability

The datasets generated and analyzed during the current study are available from the corresponding author on reasonable request.
